# High-Resolution 3D Genome Map of Brucella Chromosomes in Exponential and Stationary Phases

**DOI:** 10.1128/spectrum.04290-22

**Published:** 2023-02-27

**Authors:** Yong-Fang Huang, Lin Liu, Fei Wang, Xin-Wei Yuan, Huan-Chun Chen, Zheng-Fei Liu

**Affiliations:** a State Key Laboratory of Agricultural Microbiology, College of Veterinary Medicine, Huazhong Agricultural University, Wuhan, Hubei, China; b Wuhan Frasergen Bioinformatics Co., Ltd., Wuhan, Hubei, China; Shenzhen Bay Laboratory

**Keywords:** *Brucella*, Hi-C, three-dimensional genome, RNA-seq, short-range interaction, exponential phase, stationary phase

## Abstract

The three-dimensional (3D) genome structure of an organism or cell is highly relevant to its biological activities, but the availability of 3D genome information for bacteria, especially intracellular pathogens, is still limited. Here, we used Hi-C (high-throughput chromosome conformation capture) technology to determine the 3D chromosome structures of exponential- and stationary-phase Brucella melitensis at a 1-kb resolution. We observed that the contact heat maps of the two B. melitensis chromosomes contain a prominent diagonal and a secondary diagonal. Then, 79 chromatin interaction domains (CIDs) were detected at an optical density at 600 nm (OD_600_) of 0.4 (exponential phase), with the longest CID being 106 kb and the shortest being 12 kb. Moreover, we obtained 49,363 significant *cis*-interaction loci and 59,953 significant *trans*-interaction loci. Meanwhile, 82 CIDs of B. melitensis at an OD_600_ of 1.5 (stationary phase) were detected, with the longest CID being 94 kb and the shortest being 16 kb. In addition, 25,965 significant *cis*-interaction loci and 35,938 significant *trans*-interaction loci were obtained in this phase. Furthermore, we found that as the B. melitensis cells grew from the logarithmic to the plateau phase, the frequency of short-range interactions increased, while that of long-range interactions decreased. Finally, combined analysis of 3D genome and whole-genome transcriptome (RNA-seq) data revealed that the strength of short-range interactions in Chr1 is specifically and strongly correlated with gene expression. Overall, our study provides a global view of the chromatin interactions in the B. melitensis chromosomes, which will serve as a resource for further study of the spatial regulation of gene expression in Brucella.

**IMPORTANCE** The spatial structure of chromatin plays important roles in normal cell functions and in the regulation of gene expression. Three-dimensional genome sequencing has been performed in many mammals and plants, but the availability of such data for bacteria, especially intracellular pathogens, is still limited. Approximately 10% of sequenced bacterial genomes contain more than one replicon. However, how multiple replicons are organized within bacterial cells, how they interact, and whether these interactions help to maintain or segregate these multipartite genomes are unresolved issues. Brucella is a Gram-negative, facultative intracellular, and zoonotic bacterium. Except for Brucella suis biovar 3, Brucella species have two chromosomes. Here, we applied Hi-C technology to determine the 3D genome structures of exponential- and stationary-phase Brucella melitensis chromosomes at a 1-kb resolution. Combined analysis of the 3D genome and RNA-seq data indicated that the strength of short-range interactions in B. melitensis Chr1 is specifically and strongly correlated with gene expression. Our study provides a resource to achieve a deeper understanding of the spatial regulation of gene expression in Brucella.

## INTRODUCTION

In all organisms, the length of the genomic DNA molecule is typically several thousandfold larger than the diameter of the cell itself (1). However, chromosomes are not randomly packaged within cells but are compressed into highly organized structures, presumably to support the execution and preservation of DNA-associated processes such as replication, transcription, repair, recombination, and chromosome segregation. Chromosome conformation capture (3C) technology and extensions of its technology (4C, 5C, Hi-C [high-throughput 3C], ChIP-loop [chromatin immunoprecipitation-loop], ChIA-PET [chromatin interaction analysis by paired-end tag], etc.) have been widely applied. In 2002, the Hi-C technology was developed (2). Hi-C is an innovative technology that can be used to study spatial chromatin interactions in three-dimensional (3D) structures (2, 3). Hi-C technology involves the fixation, digestion, and ligation of chromosomes to capture fragments that contain chromatin interaction information, followed by high-throughput sequencing (2, 3). Through bioinformatics analysis, the interactions between different regions can be obtained from the spatial proximity of different regions in the whole genome, uncovering long-range regulatory elements in 3D space and revealing the mechanisms by which spatial structure regulates gene function (3). Hi-C technology has been widely used in yeasts (4), *Arabidopsis* (5, 6), fungi (7, 8), cotton (9), rice (10), maize (11), mice (12), and humans.

Currently, chromosome conformation capture technology is being applied to bacteria. Bacteria usually have a small circular DNA, and in general, most bacterial genomes range from 500 kb to ~12 Mb in size. Unlike eukaryotes, bacteria do not have a nucleus, and most of the bacterial genetic material is concentrated in a specific area of the cell, called the nucleoid. To date, spatial genome structure information has been reported for many bacteria, including Caulobacter crescentus (13–16), Bacillus subtilis (17–20), Vibrio cholerae (21, 22), Mycoplasma pneumoniae (23), Escherichia coli (1, 24), Klebsiella pneumoniae (25), *Rhizobium* (26–28), Acanthamoeba castellanii (29), *Streptomyces* (30), Pseudomonas aeruginosa (31), and the Oligo-Mouse-Microbiota community (32).

Approximately 10% of sequenced bacterial genomes contain more than one replicon (26, 33). These multipartite genomes are scattered across different bacterial phyla, including species of the *Borrelia*, *Burkholderia*, Brucella, *Rhizobium*, and *Vibrio* genera, which are mostly plant and animal symbionts or pathogens (26, 33, 34). It has been proposed that having multiple replicons allows faster genome replication and endows these bacteria with the advantage of rapid adaptation when switching hosts or environments (26, 33–35). However, multipartite genomes present challenges for genome maintenance and management. How multiple replicons are organized within bacterial cells, how they interact, and whether these interactions help maintain or segregate these multipartite genomes are pending questions. To date, the 3D genomes of a plant symbiont (*Rhizobium*) (26–28) and a human and aquatic pathogen (*Vibrio*) (21, 22) have been reported.

Brucella species are Gram-negative, facultative intracellular bacteria of the class *Alphaproteobacteria*. The pathogens can be transmitted from livestock and wildlife to humans via contact with infected animals or animal products through the respiratory, mucosal, or digestive tract. The clinical symptoms of brucellosis manifest as undulant fever, sweating, joint pain, splenomegaly, and infertility (36). Notably, Brucella does not possess classical pathogenic factors such as plasmids, exotoxins, virulence islands, or capsules that participate in infection and evade host immune surveillance (37). Except for Brucella suis biovar 3, Brucella species have two chromosomes (38). Brucella melitensis chromosome 1 (Chr1) and Chr2 are 2.12 Mb and 1.18 Mb, respectively. There are some differences in the types of genes on these two chromosomes (38). Genes on Chr1 are related to basic physiological functions, while genes on Chr2 are mainly related to adaptability and virulence (39). It has been proposed that the two chromosomes of Brucella strains evolved from a hypothetical ancestor with a single chromosome (33, 40). In addition, the GC contents of the two chromosomes are almost identical, and it can be inferred that the assimilation and stabilization of the chromosomes have already occurred (33, 41). Given that the two chromosomes coexist in a single Brucella particle, whether they interact with each other, where they interact, and the correlation of the 3D structure of the Brucella chromosomes and transcription are still unclear.

In this study, we applied Hi-C technology to determine the 3D genome structure of exponential- and stationary-phase B. melitensis chromosomes at a 1-kb resolution. The differences in the 3D genome interactions of the two B. melitensis chromosomes between the different growth states were identified. Combined analysis of the 3D genome and transcriptome sequencing (RNA-seq) data revealed that the strength of short-range interactions in B. melitensis Chr1 is specifically and strongly correlated with gene expression.

## RESULTS AND DISCUSSION

### Generation of high-resolution whole-genome contact maps for B. melitensis.

**FIG 1:**
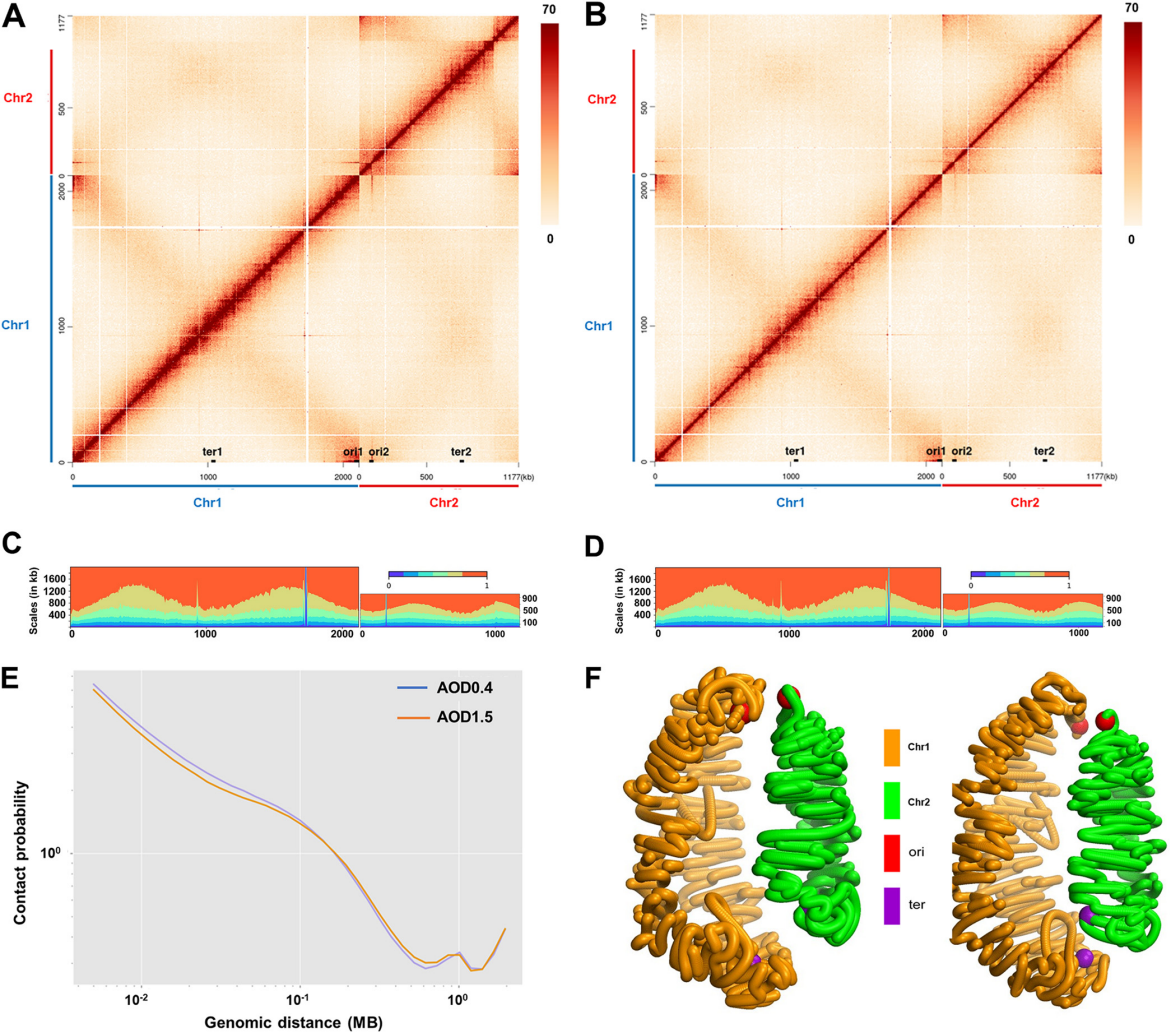
Hi-C contact maps and genome-wide organization of B. melitensis. (A and B) Interaction heat maps of B. melitensis at OD_600_s of 0.4 (early exponential [A]) and 1.5 (stationary phase [B]). Normalized Hi-C contact maps display contact frequencies for pairs of 1-kb bins across the genome of B. melitensis. The *x* and *y* axes represent the genomic coordinates of the reference genome (B. melitensis 16M [3.3 Mb]), and the color scale reflects the frequency of contacts between any two regions of the genome, from white (rare contacts) to black (frequent contacts). The positions of the two origins (ori1 and ori2) and two termini (ter1 and ter2) are indicated on the *x* axis. (C) Scalogram representation of the B. melitensis genome at an OD_600_ of 0.4. Scalograms reflect the relative tightness of the distribution of contacts made by the chromosomal regions. The colored areas above each bin represent the fraction of the total cumulated contacts made by the bin with flanking regions of increasing sizes (dark blue, 0 to 0.15; light blue, 0.15 to 0.3, etc.; red, 0.75 to 1). Compacted regions are indicated by small blue and large red areas. Loose regions are indicated by large blue and small red areas. (D) Scalogram representation at an OD_600_ of 1.5; (E) genome-wide contact decay curves at OD_600_s of 0.4 (AOD0.4) and 1.5 (AOD1.5). The *x* axis represents the relative distance between different loci on the chromosome; the *y* axis represents the interaction frequency of pairs of loci separated by this distance. (F) Three-dimensional models of the B. melitensis genome at OD_600_s of 0.4 (left) and 1.5 (right), where the brown region represents Chr1, the green region represents Chr2, the red balls are origins, and the purple balls are termini.

The 3D chromosome structure reveals the intrinsic function of genome organization in biological processes. Chromosome conformation capture combined with deep sequencing has been used previously to explore bacterial genome organization (3). To reveal the spatial structure and interaction of two chromosomes in Brucella, we applied Hi-C technology (2), which utilizes cross-linking with formaldehyde in conjunction with spatially constrained ligation to assess the average spatial proximity of genomic loci of early-exponential-phase (optical density at 600 nm [OD_600_] of 0.4 [AOD0.4]) (Fig. 1A) and stationary-phase (AOD1.5) (Fig. 1B) B. melitensis cultures. Each Hi-C experiment yields a genome-wide library of 1-kb ligation products whose frequencies reflect the 3D structure of the genome. As expected, the two chromosomes in the resulting contact maps exhibited a strong diagonal signal, reflecting frequent contacts between adjacent loci. This main diagonal represents the high-frequency interactions between DNA loci on the same arm of the chromosome (i.e., intra-arm interactions). A second, less prominent diagonal reflects lower-frequency interactions between loci on one arm of the chromosome and those on the opposite arm (i.e., interarm interactions) (23). Thus, the contact heat maps of the two B. melitensis chromosomes contain a prominent diagonal and a fuzzy secondary diagonal (Fig. 1A and B). These interactions resemble those of unichromosomal bacteria, such as C. crescentus, and of Chr2 of V. cholerae, a bacterium with a multipartite genome (21). According to the interaction matrix, the sum of interactions within a certain range upstream and downstream of each bin on the chromosome can be calculated, and the proportion of the interactions between the bin and the entire chromosome reflects the compactness of the local region on the genome. This method has been used to study the effect of a structural protein in E. coli on the degree of local spatial compactness of the genome (1). Figure 1C and D show the change in the degree of genome compaction of the two B. melitensis chromosomes at the early exponential and stationary phases. Then, contact decay curves of the genome-wide 2-kb resolution of AOD0.4 and AOD1.5 were integrated, and the differences in distances and interaction frequencies between the samples were compared and analyzed (Fig. 1E). The conversion of contact maps into 3D structures at OD_600_s of 0.4 (left) and 1.5 (right) (Fig. 1F; see Movies S1 and S2 in the supplemental material) shows that Chr1 and Chr2 fold into a helicoidal shape with its two replichores tightly interlaced. The positions of origins (red balls) and termini (purple balls) are annotated in the figures. These contact maps provide insights into the interplay of all loci in the two B. melitensis chromosomes.

### Analysis of chromatin interaction domains (CIDs) in the 3D genome of B. melitensis.

**FIG 2:**
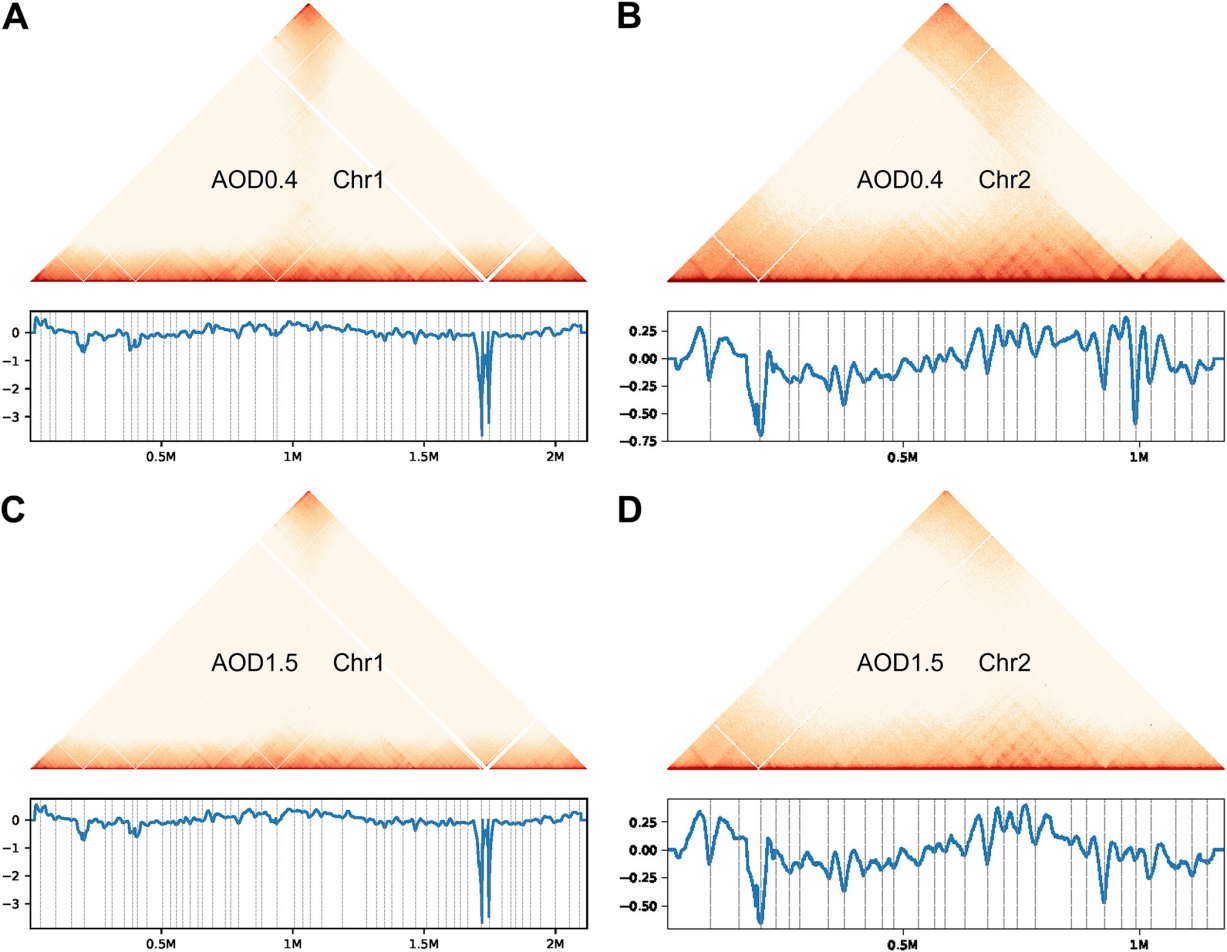
B. melitensis CID distribution at a 2-kb resolution. Panels A and C show the CID distributions of Chr1 at OD_600_s of 0.4 and 1.5, respectively. Panels B and D show the CID distributions of Chr2 at OD_600_s of 0.4 and 1.5, respectively. The *x* axis represents the position on the genome, and the *y* axis represents the insulation score. The blue color bar indicates the strength of the interaction; the larger the value is, the stronger the interaction.

Chromatin interaction domains (CIDs) refer to the high-level structures in genome space with relatively strong local interactions found on prokaryotic interaction maps (14). These are similar to the eukaryotic topologically associated domains (TADs) (42). CIDs are related to important structural and regulatory roles in bacteria. Figure 2A to D show the distribution of CIDs on chromosomes at a 2-kb resolution, including interaction heat maps, insulation scores, and CID boundaries. In B. melitensis at an OD_600_ of 0.4, 79 CID regions were detected, with the longest being 106 kb and the shortest being 12 kb. In B. melitensis at an OD_600_ of 1.5, 82 CID regions were detected, with the longest being 94 kb and the shortest being 16 kb (Table S1). This may suggest that as bacteria transition to stationary phase, the number of CIDs increases and the range becomes smaller. In order to compare the stability of the CID boundary structure between the two growth stages, we compared the CID boundaries between the samples according to the 2-kb resolution CID analysis in each sample and identified the CID boundaries that were shared between the samples and unique to each sample. Figure S1 shows that AOD0.4 (logarithmic-phase sample) shared 41 CIDs with AOD1.5 (plateau sample), while AOD0.4 and AOD1.5 had 36 and 39 unique CIDs, respectively. This result shows that the variation in CIDs during bacterial growth is relatively high.

CIDs are usually divided into border and interior regions. According to the results of CID analysis, the gene density distribution and GC content distribution of CID in B. melitensis were calculated. The results showed that there was no significant difference between the border and interior of the CIDs in terms of gene density distribution (Fig. S2A and B and Table S2) or GC content distribution (Fig. S2C and D and Table S3) at an OD_600_ of 0.4 or 1.5.

**FIG 3:**
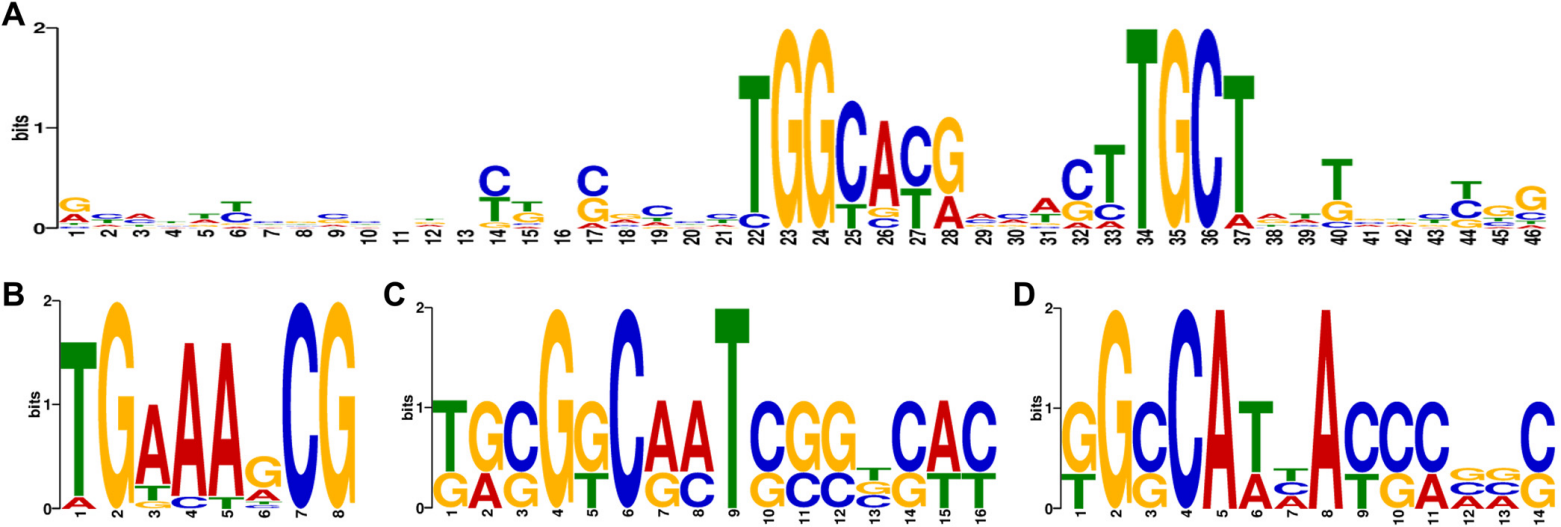
CID boundary region enrichment motif analyses. Panels A, B, C, and D represent the top four motif sequences enriched at the CID boundary.

In eukaryotes, TAD boundaries are enriched in highly expressed genes, and structural proteins such as CTCF (CCCTC-binding factor) and cohesin are usually enriched at TAD boundaries in the human genome (43). The characterization of CID or TAD boundaries is of great significance in understanding the mechanism by which the genomic topology of this species is established (44). We used FIMO software (45) to scan the motifs at the CID boundary and found that certain motifs were enriched in the boundary region. We also visualized these motif-binding sequences. The top four CID boundary enrichment motif sequences are shown in Fig. 3A to D; detailed information is shown in Table S4. These motifs are associated with transcription factors, indicating that CID boundaries are dependent on transcription.

**FIG 4:**
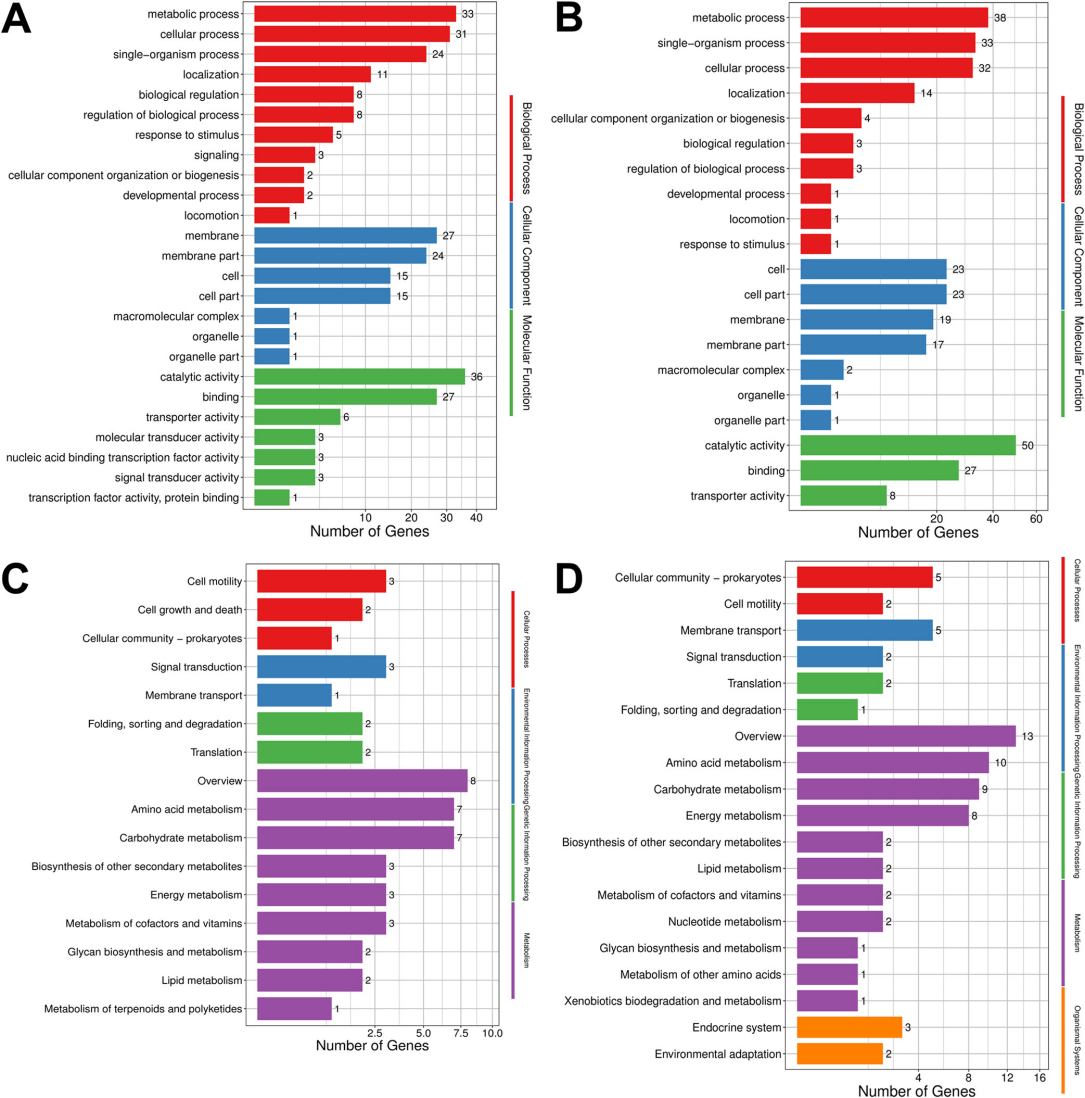
Comparison of classification and enrichment pathways of differentially expressed genes at CID boundaries at different growth stages. (A and B) GO enrichment results of the differential CID boundary regions at OD_600_s of 0.4 (A) and 1.5 (B). The horizontal axis is the number of genes. The vertical axis is the enriched GO terms. (C and D) KEGG analysis of the differential CID boundary regions at OD_600_s of 0.4 (C) and 1.5 (D). The horizontal axis indicates the number of genes, and the vertical axis indicates the pathway.

Subsequently, the gene sets located within the CID boundary in B. melitensis were subjected to Gene Ontology (GO) and Kyoto Encyclopedia of Genes and Genomes (KEGG) enrichment analysis (Fig. S3). GO analysis showed that the biological functions of the CID boundary genes were mainly enriched in metabolism and cellular processes, and their subcellular localization was mainly concentrated in the membrane and cell parts. Molecular functions were mainly enriched in catalytic activity, binding, and transport function (Fig. S3A and B). KEGG analysis showed that B. melitensis CID boundary genes were mainly involved in metabolism-related pathways (Fig. S3C and S3D). These results suggest that CID boundary genes may play important roles in B. melitensis growth and metabolism. Furthermore, to explore the functions of genes within the boundaries of the CIDs that were unique to each sample, the gene sets located within the boundaries of differential CIDs were functionally annotated (Fig. 4). Figure 4A and B show that the differential CIDs of AOD0.4 and AOD1.5 are mainly enriched in metabolic processes, and the subcellular localization of these genes is mainly enriched in the membrane and cell parts. The enriched molecular function terms are mainly related to catalytic activity, binding, and transport function. Figure 4C and D show that differential CID boundary genes are mainly involved in metabolism-related pathways.

### Analysis of interacting loci in B. melitensis at the early exponential and stationary phases.

**FIG 5:**
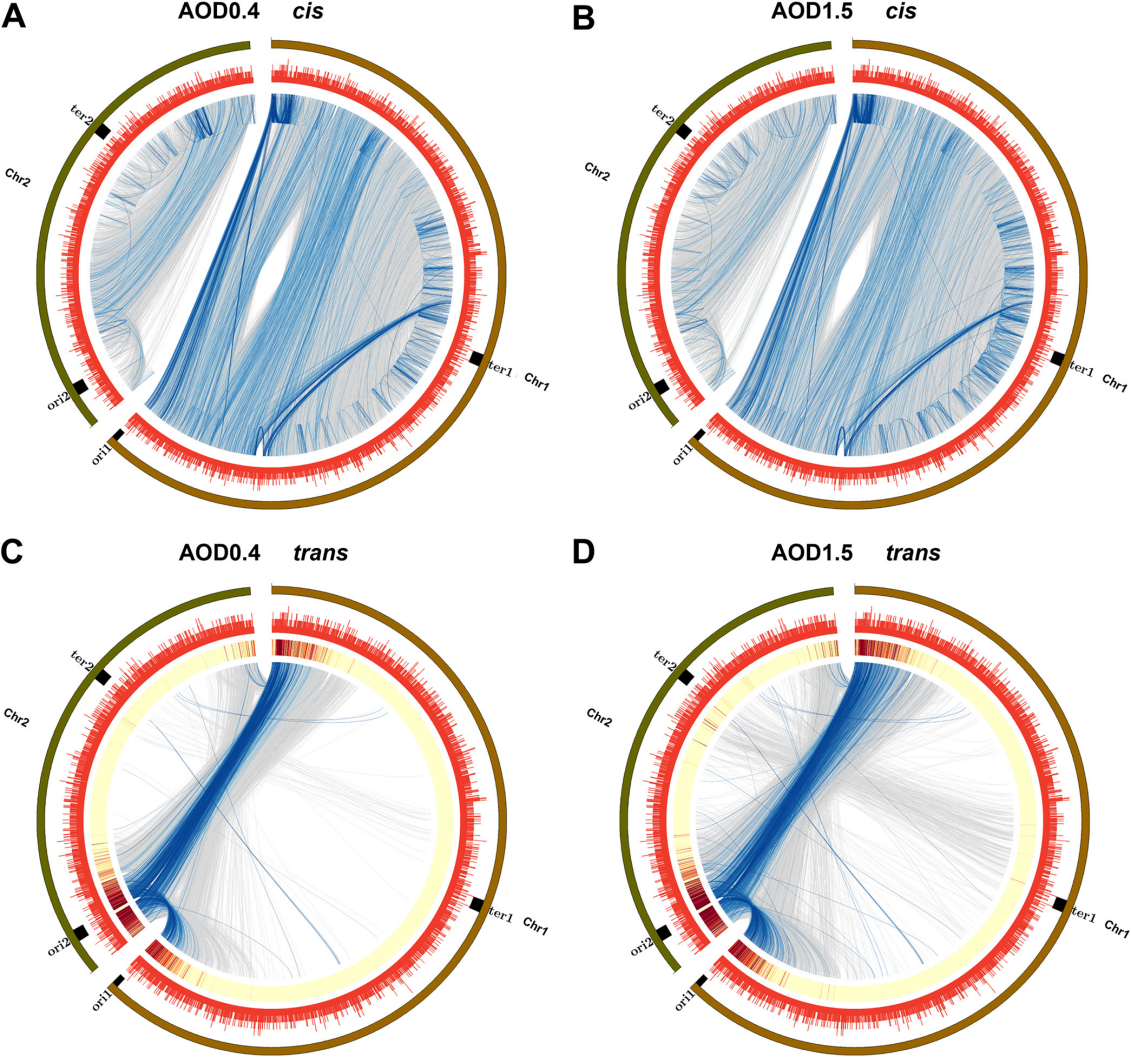
Genome-wide significant *cis*-interaction (intrachromosomal) and *trans*-interaction (interchromosomal) loci at a 1-kb resolution. (A and B) Circos plots of significant *cis*-interaction loci of B. melitensis at OD_600_s of 0.4 (A) and 1.5 (B). The outermost circle shows the chromosome name and location, the red circle shows gene density, and blue indicates significant *cis*-interaction links. The color becomes darker as the *P* value increases. (C and D) Circos diagrams of significant *trans*-interaction loci of B. melitensis at OD_600_s of 0.4 (C) and 1.5 (D). The outermost circle shows the chromosome name and location, red indicates gene density, the heat map indicates the distribution of the number of *trans*-interactions, with red to yellow indicating the number of interactions from many to a few, and blue indicates significant *trans*-interaction links. The color becomes darker as the *P* value increases.

DNA loci that are distant within the genome may become spatially close via the formation of loops, bringing different biological domains closer, which impacts transcriptional regulation (16, 46). Fit-Hi-C software (47) was employed to statistically analyze the significance of interactions between every two loci in the whole genome at a 1-kb resolution to detect the chromatin loop structure. We obtained 49,363 significant *cis*-interaction loci and 59,953 significant *trans*-interaction loci at the logarithmic growth phase (Table S5). We also obtained 25,965 significant *cis*-interaction loci and 35,938 significant *trans*-interaction loci at the stable growth phase (Table S5). Figure 5A and B show the significant *cis*-interaction loci of B. melitensis at OD_600_s of 0.4 and 1.5. Figure 5C and D show the significant *trans*-interaction loci of B. melitensis at OD_600_s of 0.4 and 1.5.

Hi-C interaction studies have shown that most interactions usually occur within chromosomes, and interchromosome interactions were long considered to represent in-matrix noise. However, in olfactory neurons, the genome forms a large number of specific *trans*-interactions in 3D structure, regulating the expression of different olfactory receptor genes (48), suggesting that *trans*-interactions may have undiscovered biological significance. In addition, recent studies have reported a number of *trans*-interactions in filamentous fungi (7). In our study, the interaction between replication origins and termini was particularly strong in the *trans*-interaction Circos plot (Fig. 5C and D), indicating that these *trans*-interactions might play important roles in the replication process of B. melitensis.

Significant *cis*-interaction loci can also be classified according to different distance ranges. B. melitensis has two chromosomes. Considering that the difference in chromosome length will affect the statistical results, we drew histograms based on distance calculations in the circular genome. Figure S4 shows that when the interaction distance on the genome is less than 200 kb, the percentage of *cis*-interactions is remarkably high regardless of the bacterial growth state. The percentage of *cis*-interactions decreased dramatically for interaction distances between 200 and 600 kb. However, there was a clear increase in the frequency of *cis*-interactions as the distance continued to increase (600 to 800 kb). When the interaction distance was longer than 800 kb, the frequency of *cis*-interactions decreased. In addition, a comparison of Fig. S4A and B shows that as the bacteria grew from logarithmic to plateau phase, the proportion of short-range interactions (<200 kb) increased, while that of long-range interactions (>200 kb) decreased.

**FIG 6:**
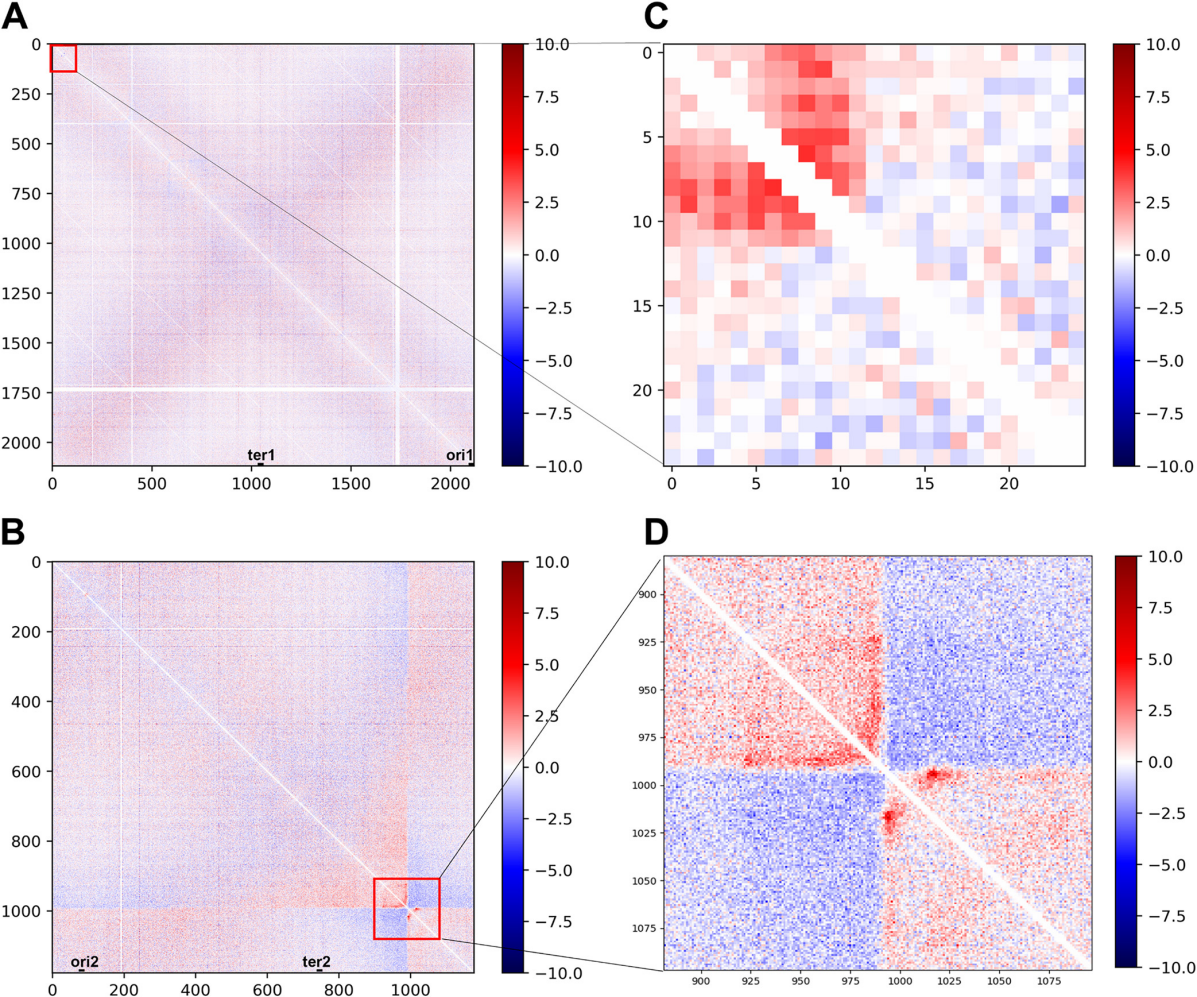
Interaction subtraction matrix of B. melitensis 3D genome structure at the early exponential and stationary phases. (A) Interaction subtraction matrix of B. melitensis Chr1 between AOD0.4 and AOD1.5 at a 1-kb resolution; (B) interaction subtraction matrix of B. melitensis Chr2 between AOD0.4 and AOD1.5 at a 1-kb resolution; (C) heat map of differentially interacting loci of the B. melitensis 3D genome on Chr1 between AOD0.4 and AOD1.5 at a 5-kb resolution; (D) heat map of differentially interacting loci of the B. melitensis 3D genome on Chr2 between AOD0.4 and AOD1.5 at a 5-kb resolution. The horizontal and vertical axes represent positions on the genome. The color bar indicates the interaction difference subtraction value. Red indicates that the interaction strength of the AOD0.4 sample at this site is greater than that of the AOD1.5 sample, while blue indicates the opposite. The larger the absolute value is, the greater the difference in the interaction strength of the site between the samples.

To visualize the differences in interactions between the early exponential and stationary phases of Brucella cultures more intuitively, an interaction subtraction matrix was utilized (49). Two heat maps representing the single chromosome 1-kb resolution interaction subtraction matrixes were obtained (Fig. 6A and B). Figure 6C and D show heat maps of the significant difference interaction subtraction matrix at 5-kb resolution within the red region in Fig. 6A and B. The main differential interactions involve B. melitensis replication origins and termini. At an OD_600_ of 0.4, the interactions between the origin of replication and other genes are strong, and these interactions are significantly reduced after the bacteria enter the stable stage. This indicates that the genes at these positions may be involved in the growth and replication of B. melitensis.

### Transcription is involved in local chromatin structure in Chr1.

**FIG 7:**
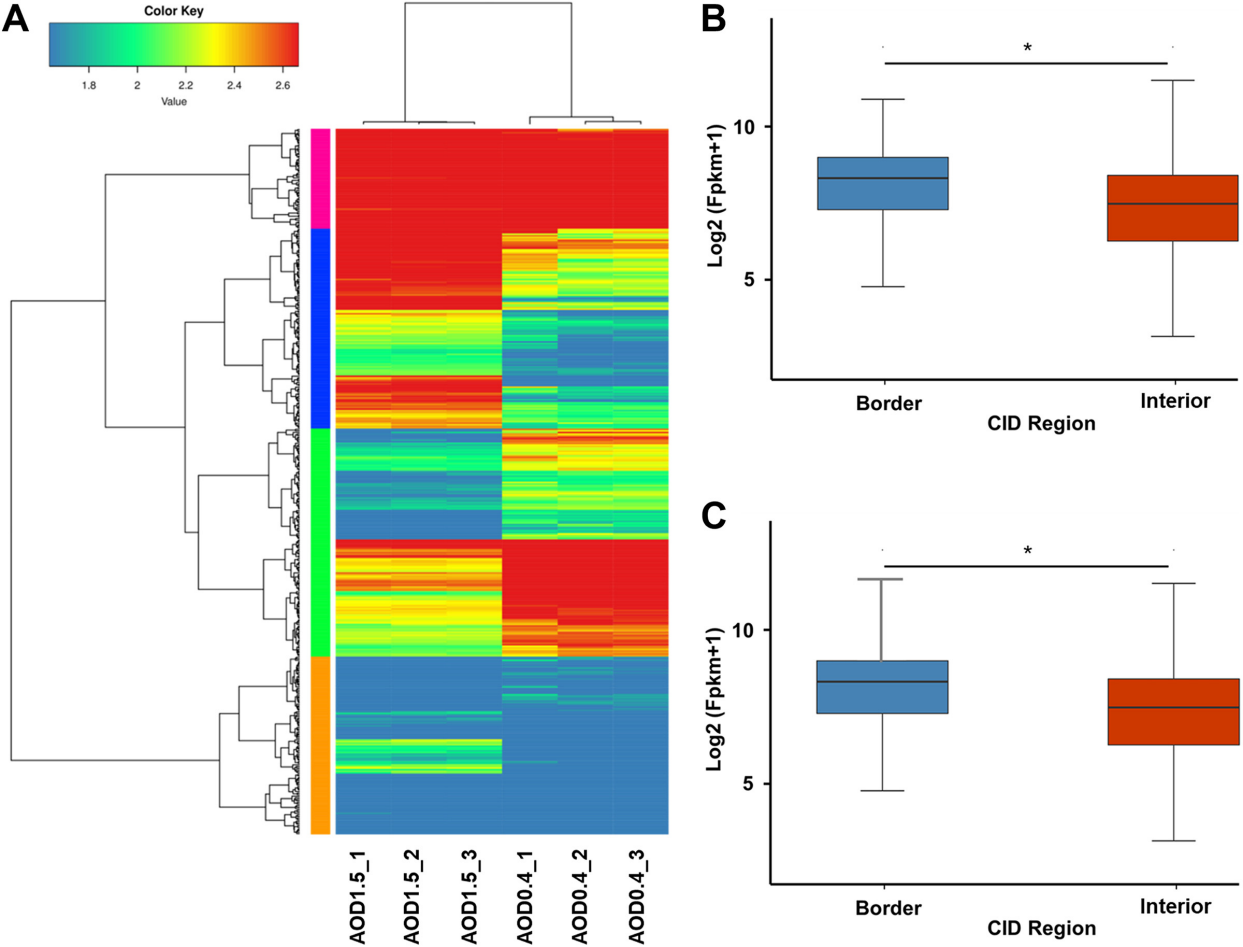
Gene expression of B. melitensis at OD_600_s of 0.4 and 1.5. (A) Differential gene expression pattern cluster diagram based on RNA-seq data; (B and C) box plots of CID border and interior expression distributions at OD_600_s of 0.4 (B) and 1.5 (C). The *x* axis indicates the CID region, including the border and interior. The *y* axis shows the log_2_ (fragments per kilobase per million [FPKM] + 1). *, *P* < 0.05.

A previous study showed that the boundary genes of CIDs are often highly expressed (1, 14). In our study, we first constructed a gene expression pattern cluster diagram for the differential genes screened by RNA-seq (Fig. 7A) of exponential- and stationary-phase B. melitensis. Then, the gene expression characteristics of the CID border and interior were detected. Figure 7B and C show that gene expression of CID border and interior regions are significantly different at both OD_600_s of 0.4 and 1.5, with CID border regions having higher gene expression levels than interior regions.

**FIG 8:**
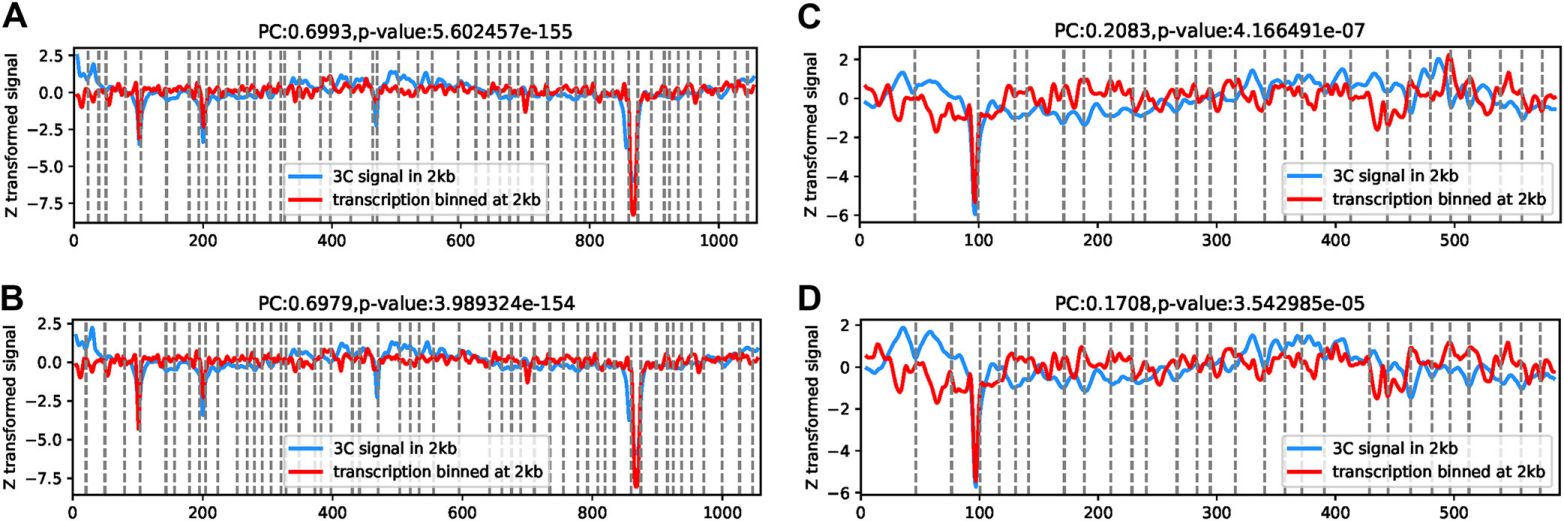
Relationship between short-range interaction strength and gene transcription in B. melitensis. (A and B) Correlation analysis of gene interaction strength and gene expression on Chr1 at OD_600_s of 0.4 (A) and 1.5 (B) at a 2-kb resolution; (C and D) correlation analysis of gene interaction strength and gene expression level on Chr2 at OD_600_s of 0.4 (C) and 1.5 (D) at a 2-kb resolution. The horizontal axis represents the position on the genome, and the unit is a bin. The longitudinal axis represents the normalized signal value strength. The blue line represents the 3C signal in 2-kb bins, which reflects the distribution of the short-distance interaction intensity on the chromosome at 2-kb resolution, and the signal value has been standardized. The red lines indicate the transcription binned at a 2-kb resolution. The distribution on the chromosomes and the signal values were standardized. The dotted line indicates the CID boundary. PC, Pearson correlation coefficient. The *P* value indicates the credibility of the correlation.

Moreover, it was reported that transcription level, transcription rate, and transcript length drive the formation of chromosomal interaction domain boundaries (14, 15). In our study, to verify the correlation of transcription and the 3D genome structure in B. melitensis, scatterplots between 3C signal and the gene transcription level of AOD0.4 and AOD1.5 were created as shown in Fig. S5A and B, respectively. The results showed a positive correlation. Then, we analyzed the curves of the short-distance interaction strength and gene expression of the two chromosomes under different culture conditions. Figure 8A and B show the strong correlation between the transcription levels and short-range contact frequency in B. melitensis Chr1 (Pearson correlation [PC] of >0.6). Remarkably, these correlations were also observed in contact maps of E. coli and B. subtilis generated by different laboratories using different enzymes and cross-linking conditions (1). However, Chr2 exhibits a weak correlation (PC of <0.21) between transcription level and short-range interaction strength (Fig. 8C and D), similar to the pattern observed in M. pneumoniae and C. crescentus (1). These results imply the existence of transcription-induced constraints in B. melitensis Chr1 that favor interactions between neighboring loci.

The underlying mechanisms between transcription and short-range contacts remain to be deciphered. Nucleoid-associated proteins (NAPs) are a class of nucleic acid-binding basic proteins present in prokaryotic cells that can bind to bacterial chromosomes to form compact structures—that is, nucleoids. Local binding of NAP H-NS in E. coli inhibits short-range interactions, whereas HU and Fis promote long-range contacts in different ways (1). A recent study found that the transcription factor Rok forms large nucleoprotein complexes in B. subtilis, and the complexes interact robustly with each other over long distances. These Rok-dependent long-range interactions lead to the formation of anchored chromosome loops that spatially isolate large sections of DNA (50). However, whether these NAPs play important roles in the changes in the 3D genome during the growth of B. melitensis and whether this mechanism is applicable to B. melitensis remain to be studied further.

### Conclusions.

This study provides whole-genome contact heat maps of the exponential- and stationary-phase B. melitensis chromosomes at a 1-kb resolution. In total, 79 CID regions, 49,363 significant *cis*-interaction loci, and 59,953 significant *trans*-interaction loci were detected in B. melitensis at the logarithmic growth phase. Meanwhile, a total of 82 CID regions, 25,965 significant *cis*-interaction loci, and 35,938 significant *trans*-interaction loci were obtained for B. melitensis in the stable growth phase. Furthermore, combined analysis of 3D genome and RNA-seq data revealed that the strength of short-range interactions in B. melitensis Chr1 is specifically and strongly correlated with gene expression. Taken together, the results of our study provide a model to explain certain differences of Brucella in different growth periods from a 3D genome perspective and will serve as a resource for deeper study of the spatial regulation of gene expression in Brucella, which will contribute to further understanding of Brucella’s growth and pathogenesis.

## MATERIALS AND METHODS

### Bacterial strains, media, and growth conditions.

Bacterial culture was performed as reported previously (51, 52). Briefly, B. melitensis 16M was grown at 37°C in either tryptic soy broth (TSB) (BD; no. 211825) or tryptic soy agar (TSA) (BD; no. 236950). The experiments on B. melitensis were conducted in a biosafety level 3 laboratory.

### Chromosome conformation capture with deep sequencing.

Hi-C (chromosome conformation capture with deep sequencing) experiments were performed as described previously, with slight modifications (7, 14). B. melitensis cells were grown in TSB, and bacterial growth was measured via optical density at 600 nm (OD_600_). Bacterial cultures were collected when the OD_600_ value reached 0.4 (exponential phase) or 1.5 (stationary phase). To fix the long-range DNA interactions, bacterial DNA was cross-linked with 3% formaldehyde (methanol free; Pierce) for 30 min at room temperature and another 30 min at 4°C. The reaction was stopped with 375 mM glycine for 15 min at 4°C, and the cells were washed with SET buffer (20 mM Tris [pH 8.0], 25 mM EDTANa_2_·2H_2_O [pH 8.0], 75 mM NaCl) by centrifugation at 2,000 × *g* for 5 min at 4°C. The supernatant was discarded, and the cells were resuspended thoroughly in 3 mL SET buffer. Aliquots of bacteria were frozen at −80°C.

Hi-C library construction and sequencing were carried out as described previously (7). Briefly, the genomic DNA from the bacteria was cross-linked for 1 h with 3% formaldehyde at 4°C and quenched with glycine at a final concentration of 0.375 M for 15 min. The cross-linked cells were lysed by incubation with lysozyme at 37°C for 10 min. Endogenous nucleases were inactivated with 0.5% SDS, and chromatin DNA was digested with 50 U Sau3AI (New England BioLabs, Inc.). Restriction fragment ends were labeled with biotinylated cytosine nucleotides by biotin-14-dCTP (TriLINK), followed by ligation using 50 U T4 DNA ligase (New England BioLabs, Inc., Ipswich, MA). After reversing the cross-links, the ligated DNA was extracted through using a QIAamp DNA minikit (Qiagen) according to the manufacturers’ instructions. Purified DNA was sheared to a length of 300 to 500 bp and was further blunt-end repaired and A-tailed, with adaptor added, using the NEBNext Ultra II DNA library prep kit for Illumina (NEB, catalog no. E7645S). Point ligation junctions were pulled down with Dynabeads MyOne conjugated with streptavidin C1 (Thermo Fisher), followed by PCR amplification. Then, the PCR product was subjected to paired-end sequencing by BGI on the MGISEQ-2000 platform with PE150. Two biological replicates were used for each sample.

### RNA isolation.

RNA isolation was performed as described previously (51, 52). Briefly, B. melitensis 16M was grown at 37°C and 180 rpm to an OD_600_ of 0.6 to ~0.8. The growth of the bacterial culture (5 mL per sample) was stopped by adding 0.625 mL of prechilled stop solution (5% phenol–95% ethanol). Cells were harvested by centrifugation for 10 min at 6,000 × *g*, and the resulting pellet was stored at −80°C. Frozen cell pellets were thawed on ice and resuspended in 100 μL per pellet of TE buffer (10 mM Tris-HCl, 1 mM EDTA [pH 8.0]). Total RNA was extracted using TRIzol reagent (Invitrogen, Carlsbad, CA, USA). The RNA samples were then subjected to treatment with DNase I (TaKaRa, Dalian, China) to remove genomic DNA. The concentration and quality of each sample were evaluated using a NanoDrop instrument (Thermo Fisher Scientific, Waltham, MA, USA) and agarose gel electrophoresis.

### RNA-seq.

RNA library construction and sequencing were performed as described (1). Briefly, total RNA was stabilized *in vivo* with RNAprotect bacterial reagent (Qiagen) and purified with an RNeasy Protect bacterial minikit (Qiagen). rRNA was depleted with a RiboZero-rRNA removal kit for bacteria (Epicenter). Libraries were prepared according to the instructions accompanying the Illumina TruSeq SBS Transcription kit.

### Hi-C data analysis.

The sequenced raw reads were filtered and quality evaluated using Trimmomatic (53) and FastQC software (54) with the default parameters to generate clean reads. The clean reads obtained were mapped onto the genome of the B. melitensis 16M strain, and valid paired reads were generated by the hiclib pipeline. The reliability of the Hi-C data from the two replicates was assessed with Genome DISCO. Chromatin interaction domains (CIDs) were identified by calculating the insulation score values using the software cworld-dekker. The intra- and interchromosomal interactions at the resolution of 1-kb bins were selected by Fit-Hi-C software (47), with thresholds of a *P* value of ≤0.01, false-discovery rate (*q*) value of ≤0.01, and contact count of >2. Motif scanning was carried out in the JASPAR database (http://jaspar.genereg.net) using MEME Suite (http://meme-suite.org).

### Establishment of 3D genomic model of B. melitensis.

The 3D model of the B. melitensis genome was predicted from Hi-C data by the PASTIS software using a 1-kb resolution matrix with the multidimensional scaling (MDS) module (55). In the 3D model of B. melitensis based on the obtained Hi-C data, the origin and terminus regions were annotated with Centurion software (56).

### Data availability.

The Hi-C and RNA-seq data of the B. melitensis 16M strain generated in this study have been submitted to the NCBI BioProject database (https://www.ncbi.nlm.nih.gov/bioproject/) under accession no. PRJNA918778 and PRJNA916855, respectively.

## References

[1] Lioy VS, Cournac A, Marbouty M, Duigou S, Mozziconacci J, Espéli O, Boccard F, Koszul R. 2018. Multiscale structuring of the *E. coli* chromosome by nucleoid-associated and condensin proteins. Cell 172:771–783.e18. doi:10.1016/j.cell.2017.12.027. 29358050

[2] Dekker J, Rippe K, Dekker M, Kleckner N. 2002. Capturing chromosome conformation. Science 295:1306–1311. doi:10.1126/science.1067799. 11847345

[3] Lieberman-Aiden E, van Berkum NL, Williams L, Imakaev M, Ragoczy T, Telling A, Amit I, Lajoie BR, Sabo PJ, Dorschner MO, Sandstrom R, Bernstein B, Bender MA, Groudine M, Gnirke A, Stamatoyannopoulos J, Mirny LA, Lander ES, Dekker J. 2009. Comprehensive mapping of long-range interactions reveals folding principles of the human genome. Science 326:289–293. doi:10.1126/science.1181369. 19815776 PMC2858594

[4] Kim S, Liachko I, Brickner DG, Cook K, Noble WS, Brickner JH, Shendure J, Dunham MJ. 2017. The dynamic three-dimensional organization of the diploid yeast genome. eLife 6:e23623. doi:10.7554/eLife.23623. 28537556 PMC5476426

[5] Yadav VK, Santos-González J, Köhler C. 2021. INT-Hi-C reveals distinct chromatin architecture in endosperm and leaf tissues of *Arabidopsis*. Nucleic Acids Res 49:4371–4385. doi:10.1093/nar/gkab191. 33744975 PMC8096224

[6] Di Stefano M, Nützmann H-W, Marti-Renom MA, Jost D. 2021. Polymer modelling unveils the roles of heterochromatin and nucleolar organizing regions in shaping 3D genome organization in *Arabidopsis thaliana*. Nucleic Acids Res 49:1840–1858. doi:10.1093/nar/gkaa1275. 33444439 PMC7913674

[7] Li C-X, Liu L, Zhang T, Luo X-M, Feng J-X, Zhao S. 2022. Three-dimensional genome map of the filamentous fungus *Penicillium oxalicum*. Microbiol Spectr 10:e02121-21. doi:10.1128/spectrum.02121-21. 35499317 PMC9241887

[8] Yuan X-L, Zhang C-S, Kong F-Y, Zhang Z-F, Wang F-L. 2021. Genome analysis of *Phytophthora nicotianae* JM01 provides insights into its pathogenicity mechanisms. Plants 10:1620. doi:10.3390/plants10081620. 34451665 PMC8400872

[9] Wang M, Wang P, Lin M, Ye Z, Li G, Tu L, Shen C, Li J, Yang Q, Zhang X. 2018. Evolutionary dynamics of 3D genome architecture following polyploidization in cotton. Nat Plants 4:90–97. doi:10.1038/s41477-017-0096-3. 29379149

[10] Liu C, Cheng Y-J, Wang J-W, Weigel D. 2017. Prominent topologically associated domains differentiate global chromatin packing in rice from *Arabidopsis*. Nat Plants 3:742–748. doi:10.1038/s41477-017-0005-9. 28848243

[11] Dong P, Tu X, Chu P-Y, Lü P, Zhu N, Grierson D, Du B, Li P, Zhong S. 2017. 3D chromatin architecture of large plant genomes determined by local A/B compartments. Mol Plant 10:1497–1509. doi:10.1016/j.molp.2017.11.005. 29175436

[12] Du Z, Zheng H, Huang B, Ma R, Wu J, Zhang X, He J, Xiang Y, Wang Q, Li Y, Ma J, Zhang X, Zhang K, Wang Y, Zhang MQ, Gao J, Dixon JR, Wang X, Zeng J, Xie W. 2017. Allelic reprogramming of 3D chromatin architecture during early mammalian development. Nature 547:232–235. doi:10.1038/nature23263. 28703188

[13] Umbarger MA, Toro E, Wright MA, Porreca GJ, Baù D, Hong S-H, Fero MJ, Zhu LJ, Marti-Renom MA, McAdams HH, Shapiro L, Dekker J, Church GM. 2011. The three-dimensional architecture of a bacterial genome and its alteration by genetic perturbation. Mol Cell 44:252–264. doi:10.1016/j.molcel.2011.09.010. 22017872 PMC3874842

[14] Le TB, Imakaev MV, Mirny LA, Laub MT. 2013. High-resolution mapping of the spatial organization of a bacterial chromosome. Science 342:731–734. doi:10.1126/science.1242059. 24158908 PMC3927313

[15] Le TB, Laub MT. 2016. Transcription rate and transcript length drive formation of chromosomal interaction domain boundaries. EMBO J 35:1582–1595. doi:10.15252/embj.201593561. 27288403 PMC4946140

[16] Messelink JJ, van Teeseling MC, Janssen J, Thanbichler M, Broedersz CP. 2021. Learning the distribution of single-cell chromosome conformations in bacteria reveals emergent order across genomic scales. Nat Commun 12:1963. doi:10.1038/s41467-021-22189-x. 33785756 PMC8010069

[17] Marbouty M, Le Gall A, Cattoni DI, Cournac A, Koh A, Fiche J-B, Mozziconacci J, Murray H, Koszul R, Nollmann M. 2015. Condensin-and replication-mediated bacterial chromosome folding and origin condensation revealed by Hi-C and super-resolution imaging. Mol Cell 59:588–602. doi:10.1016/j.molcel.2015.07.020. 26295962

[18] Brandão HB, Ren Z, Karaboja X, Mirny LA, Wang X. 2021. DNA-loop-extruding SMC complexes can traverse one another *in vivo*. Nat Struct Mol Biol 28:642–651. doi:10.1038/s41594-021-00626-1. 34312537 PMC8878250

[19] Wang X, Hughes AC, Brandão HB, Walker B, Lierz C, Cochran JC, Oakley MG, Kruse AC, Rudner DZ. 2018. *In vivo* evidence for ATPase-dependent DNA translocation by the *Bacillus subtilis* SMC condensin complex. Mol Cell 71:841–847.e5. doi:10.1016/j.molcel.2018.07.006. 30100265 PMC6591583

[20] Wang X, Le TB, Lajoie BR, Dekker J, Laub MT, Rudner DZ. 2015. Condensin promotes the juxtaposition of DNA flanking its loading site in *Bacillus subtilis*. Genes Dev 29:1661–1675. doi:10.1101/gad.265876.115. 26253537 PMC4536313

[21] Val M-E, Marbouty M, de Lemos Martins F, Kennedy SP, Kemble H, Bland MJ, Possoz C, Koszul R, Skovgaard O, Mazel D. 2016. A checkpoint control orchestrates the replication of the two chromosomes of *Vibrio cholerae*. Sci Adv 2:e1501914. doi:10.1126/sciadv.1501914. 27152358 PMC4846446

[22] Yin M, Ye B, Jin Y, Liu L, Zhang Y, Li P, Wang Y, Li Y, Han Y, Shen W, Zhao Z. 2020. Changes in *Vibrio natriegens* growth under simulated microgravity. Front Microbiol 11:2040. doi:10.3389/fmicb.2020.02040. 32983034 PMC7483581

[23] Trussart M, Yus E, Martinez S, Baù D, Tahara YO, Pengo T, Widjaja M, Kretschmer S, Swoger J, Djordjevic S, Turnbull L, Whitchurch C, Miyata M, Marti-Renom MA, Lluch-Senar M, Serrano L. 2017. Defined chromosome structure in the genome-reduced bacterium *Mycoplasma pneumoniae*. Nat Commun 8:14665. doi:10.1038/ncomms14665. 28272414 PMC5344976

[24] Wasim A, Gupta A, Mondal J. 2021. A Hi-C data-integrated model elucidates *E. coli* chromosome’s multiscale organization at various replication stages. Nucleic Acids Res 49:3077–3091. doi:10.1093/nar/gkab094. 33660781 PMC8034658

[25] Wang Y, Shen W, Yin M, Huang W, Ye B, Li P, Shi S, Bai G, Guo X, Jin Y, Lin K, Zhang Y, Jiang Y, Wang J, Han Y, Zhao Z. 2022. Changes in higher-order chromosomal structure of *Klebsiella pneumoniae* under simulated microgravity. Front Microbiol 13:879321. doi:10.3389/fmicb.2022.879321. 35711756 PMC9197264

[26] Ren Z, Liao Q, Karaboja X, Barton IS, Schantz EG, Mejia-Santana A, Fuqua C, Wang X. 2022. Conformation and dynamic interactions of the multipartite genome in *Agrobacterium tumefaciens*. Proc Natl Acad Sci USA 119:e2115854119. doi:10.1073/pnas.2115854119. 35101983 PMC8833148

[27] Ren Z, Liao Q, Barton IS, Wiesler EE, Fuqua C, Wang X. 2022. Centromere interactions promote the maintenance of the multipartite genome in *Agrobacterium tumefaciens*. mBio 13:e00508-22. doi:10.1128/mbio.00508-22. 35536004 PMC9239152

[28] Wu Z, Chen H, Pan Y, Feng H, Fang D, Yang J, Wang Y, Yang J, Sahu SK, Liu J, Xing Y, Wang X, Liu M, Luo X, Gao P, Li L, Liu Z, Yang H, Liu X, Xu X, Liu H, Wang E. 2022. Genome of *Hippophae rhamnoides* provides insights into a conserved molecular mechanism in actinorhizal and rhizobial symbioses. New Phytol 235:276–291. doi:10.1111/nph.18017. 35118662

[29] Matthey-Doret C, Colp MJ, Escoll P, Thierry A, Moreau P, Curtis B, Sahr T, Sarrasin M, Gray MW, Lang BF, Archibald JM, Buchrieser C, Koszul R. 2022. Chromosome-scale assemblies of *Acanthamoeba castellanii* genomes provide insights into *Legionella pneumophila* infection-related chromatin reorganization. Genome Res 32:1698–1710. doi:10.1101/gr.276375.121. 36109147 PMC9528979

[30] Lioy VS, Lorenzi J-N, Najah S, Poinsignon T, Leh H, Saulnier C, Aigle B, Lautru S, Thibessard A, Lespinet O, Leblond P, Jaszczyszyn Y, Gorrichon K, Varoquaux N, Junier I, Boccard F, Pernodet J-L, Bury-Moné S. 2021. Dynamics of the compartmentalized *Streptomyces* chromosome during metabolic differentiation. Nat Commun 12:5221. doi:10.1038/s41467-021-25462-1. 34471117 PMC8410849

[31] Lioy VS, Junier I, Lagage V, Vallet I, Boccard F. 2020. Distinct activities of bacterial condensins for chromosome management in *Pseudomonas aeruginosa*. Cell Rep 33:108344. doi:10.1016/j.celrep.2020.108344. 33147461

[32] Lamy-Besnier Q, Koszul R, Debarbieux L, Marbouty M. 2021. Closed and high-quality bacterial genome sequences of the Oligo-Mouse-Microbiota community. Microbiol Resour Announc 10:e01396-20. doi:10.1128/MRA.01396-20. 33927045 PMC8086220

[33] Dicenzo GC, Finan TM. 2017. The divided bacterial genome: structure, function, and evolution. Microbiol Mol Biol Rev 81:e00019-17. doi:10.1128/MMBR.00019-17. 28794225 PMC5584315

[34] Misra HS, Maurya GK, Kota S, Charaka VK. 2018. Maintenance of multipartite genome system and its functional significance in bacteria. J Genet 97:1013–1038. doi:10.1007/s12041-018-0969-x. 30262715

[35] Jha JK, Baek JH, Venkova-Canova T, Chattoraj DK. 2012. Chromosome dynamics in multichromosome bacteria. Biochim Biophys Acta 1819:826–829. doi:10.1016/j.bbagrm.2012.01.012. 22306663 PMC3348396

[36] Roop RM, Barton IS, Hopersberger D, Martin DW. 2021. Uncovering the hidden credentials of *Brucella* virulence. Microbiol Mol Biol Rev 85:e00021-19. doi:10.1128/MMBR.00021-19. 33568459 PMC8549849

[37] Jiao H, Zhou Z, Li B, Xiao Y, Li M, Zeng H, Guo X, Gu G. 2021. The mechanism of facultative intracellular parasitism of *Brucella*. Int J Mol Sci 22:3673. doi:10.3390/ijms22073673. 33916050 PMC8036852

[38] Michaux S, Paillisson J, Carles-Nurit M-J, Bourg G, Allardet-Servent A, Ramuz M. 1993. Presence of two independent chromosomes in the *Brucella melitensis* 16M genome. J Bacteriol 175:701–705. doi:10.1128/jb.175.3.701-705.1993. 8423146 PMC196208

[39] Michaux-Charachon S, Bourg G, Jumas-Bilak E, Guigue-Talet P, Allardet-Servent A, O'Callaghan D, Ramuz M. 1997. Genome structure and phylogeny in the genus *Brucella*. J Bacteriol 179:3244–3249. doi:10.1128/jb.179.10.3244-3249.1997. 9150220 PMC179103

[40] Jumas-Bilak E, Michaux-Charachon S, Bourg G, O'Callaghan D, Ramuz M. 1998. Differences in chromosome number and genome rearrangements in the genus *Brucella*. Mol Microbiol 27:99–106. doi:10.1046/j.1365-2958.1998.00661.x. 9466259

[41] DelVecchio VG, Kapatral V, Redkar RJ, Patra G, Mujer C, Los T, Ivanova N, Anderson I, Bhattacharyya A, Lykidis A, Reznik G, Jablonski L, Larsen N, D'Souza M, Bernal A, Mazur M, Goltsman E, Selkov E, Elzer PH, Hagius S, O'Callaghan D, Letesson J-J, Haselkorn R, Kyrpides N, Overbeek R. 2002. The genome sequence of the facultative intracellular pathogen *Brucella melitensis*. Proc Natl Acad Sci USA 99:443–448. doi:10.1073/pnas.221575398. 11756688 PMC117579

[42] Dixon JR, Selvaraj S, Yue F, Kim A, Li Y, Shen Y, Hu M, Liu JS, Ren B. 2012. Topological domains in mammalian genomes identified by analysis of chromatin interactions. Nature 485:376–380. doi:10.1038/nature11082. 22495300 PMC3356448

[43] Gabriele M, Brandão HB, Grosse-Holz S, Jha A, Dailey GM, Cattoglio C, Hsieh T-HS, Mirny L, Zechner C, Hansen AS. 2022. Dynamics of CTCF and cohesin mediated chromatin looping revealed by live-cell imaging. Science 376:496–501. doi:10.1126/science.abn6583. 35420890 PMC9069445

[44] Luo H, Zhu G, Eshelman MA, Fung TK, Lai Q, Wang F, Zeisig BB, Lesperance J, Ma X, Chen S, Cesari N, Cogle C, Chen B, Xu B, Yang F-C, So CWE, Qiu Y, Xu M, Huang S. 2022. HOTTIP-dependent R-loop formation regulates CTCF boundary activity and TAD integrity in leukemia. Mol Cell 82:833–851.e11. doi:10.1016/j.molcel.2022.01.014. 35180428 PMC8985430

[45] Grant CE, Bailey TL, Noble WS. 2011. FIMO: scanning for occurrences of a given motif. Bioinformatics 27:1017–1018. doi:10.1093/bioinformatics/btr064. 21330290 PMC3065696

[46] Feng Y, Cai L, Hong W, Zhang C, Tan N, Wang M, Wang C, Liu F, Wang X, Ma J, Gao C, Kumar M, Mo Y, Geng Q, Luo C, Lin Y, Chen H, Wang S-Y, Watson MJ, Jegga AG, Pedersen RA, Fu J-d, Wang ZV, Fan G-C, Sadayappan S, Wang Y, Pauklin S, Huang F, Huang W, Jiang L. 2022. Rewiring of 3D chromatin topology orchestrates transcriptional reprogramming and the development of human dilated cardiomyopathy. Circulation 145:1663–1683. doi:10.1161/CIRCULATIONAHA.121.055781. 35400201 PMC9251830

[47] Ay F, Bailey TL, Noble WS. 2014. Statistical confidence estimation for Hi-C data reveals regulatory chromatin contacts. Genome Res 24:999–1011. doi:10.1101/gr.160374.113. 24501021 PMC4032863

[48] Monahan K, Horta A, Lomvardas S. 2019. LHX2-and LDB1-mediated trans interactions regulate olfactory receptor choice. Nature 565:448–453. doi:10.1038/s41586-018-0845-0. 30626972 PMC6436840

[49] Crane E, Bian Q, McCord RP, Lajoie BR, Wheeler BS, Ralston EJ, Uzawa S, Dekker J, Meyer BJ. 2015. Condensin-driven remodelling of X chromosome topology during dosage compensation. Nature 523:240–244. doi:10.1038/nature14450. 26030525 PMC4498965

[50] Dugar G, Hofmann A, Heermann DW, Hamoen LW. 2022. A chromosomal loop anchor mediates bacterial genome organization. Nat Genet 54:194–201. doi:10.1038/s41588-021-00988-8. 35075232

[51] Liu F, Zheng K, Chen H-C, Liu Z-F. 2018. Capping-RACE: a simple, accurate, and sensitive 5′ RACE method for use in prokaryotes. Nucleic Acids Res 46:e129. doi:10.1093/nar/gky739. 30107543 PMC6265449

[52] Liu F, Huang Y-F, Wu C-X, Duan L-C, Chen H-C, Liu Z-F. 2022. Genome-wide transcription start site mapping in the facultative intracellular pathogen *Brucella melitensis* by Capping-seq. Gene 844:146827. doi:10.1016/j.gene.2022.146827. 35995114

[53] Bolger AM, Lohse M, Usadel B. 2014. Trimmomatic: a flexible trimmer for Illumina sequence data. Bioinformatics 30:2114–2120. doi:10.1093/bioinformatics/btu170. 24695404 PMC4103590

[54] Wingett SW, Andrews S. 2018. FastQ Screen: a tool for multi-genome mapping and quality control. F1000Res 7:1338. doi:10.12688/f1000research.15931.2. 30254741 PMC6124377

[55] Varoquaux N, Ay F, Noble WS, Vert J-P. 2014. A statistical approach for inferring the 3D structure of the genome. Bioinformatics 30:i26–i33. doi:10.1093/bioinformatics/btu268. 24931992 PMC4229903

[56] Varoquaux N, Liachko I, Ay F, Burton JN, Shendure J, Dunham MJ, Vert J-P, Noble WS. 2015. Accurate identification of centromere locations in yeast genomes using Hi-C. Nucleic Acids Res 43:5331–5339. doi:10.1093/nar/gkv424. 25940625 PMC4477656

