# Supplementary material for: High-Resolution 3D Genome Map of Brucella Chromosomes in Exponential and Stationary Phases
Source: Microbiol Spectr. 2023 Feb 27;11(2):e04290-22. doi: 10.1128/spectrum.04290-22 (PMC10100373; doi:10.1128/spectrum.04290-22)
Supplement: Supplemental file 1 — Supplemental material. Download spectrum.04290-22-s0001.pdf, PDF file, 0.8 MB [file spectrum.04290-22-s0001.pdf]

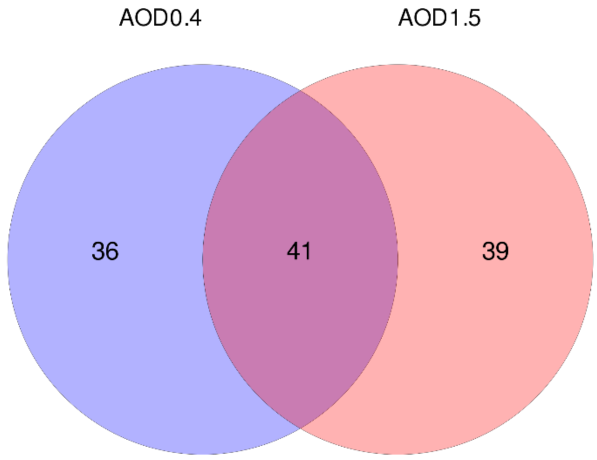

Figure S1 Venn diagram of CID boundaries between OD_{600}=0.4 and 1.5. The number in the figure is the number of CID boundaries located in the region.

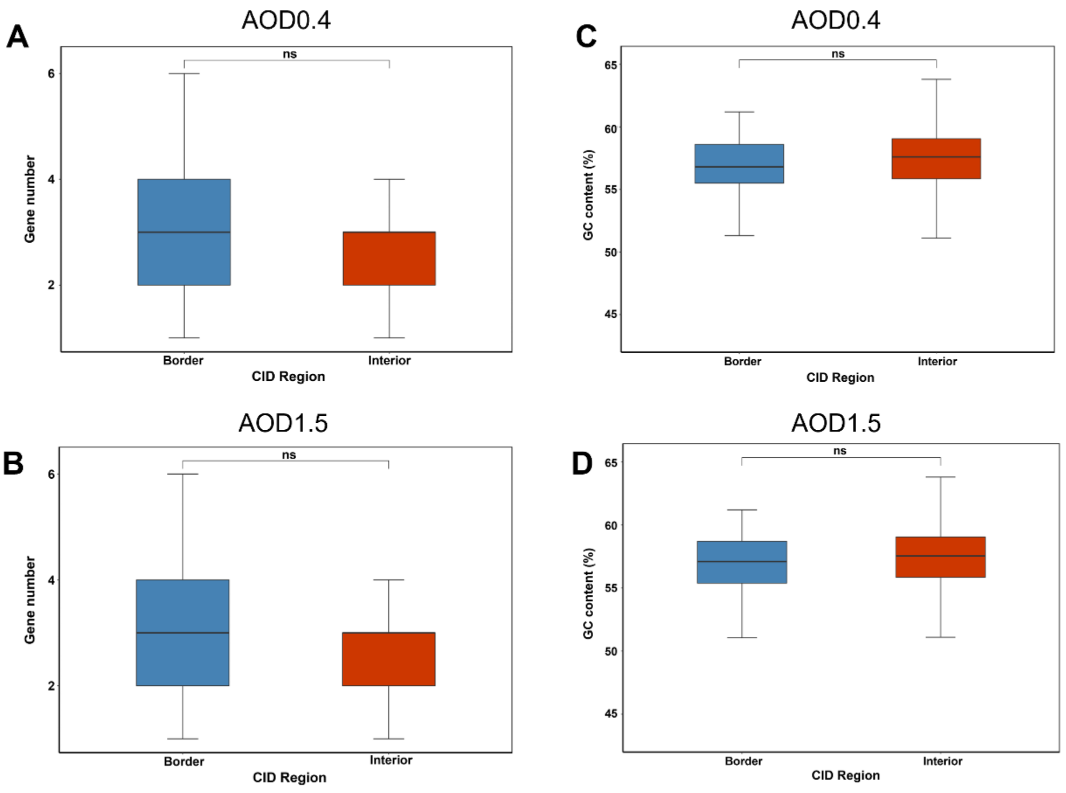

Figure S2 Gene distribution of CIDs in *B. melitensis*. (**A**), (**B**) Gene distribution of CID regions at OD_{600}=0.4 and 1.5, respectively. The x-axes represent the CID region,

comprising the border and interior. The y-axes represent the number of genes within the CID region. **(C), (D)** GC content distribution of CID regions under OD_{600}=0.4 and 1.5, respectively. The x-axes represent the CID regions; the y-axes represent the GC content of the CID regions. The box plot for each region shows five statistical parameters (from top to bottom: maximum, upper quartile, median, lower quartile, and minimum value) after removing the outliers. ns, not significant.

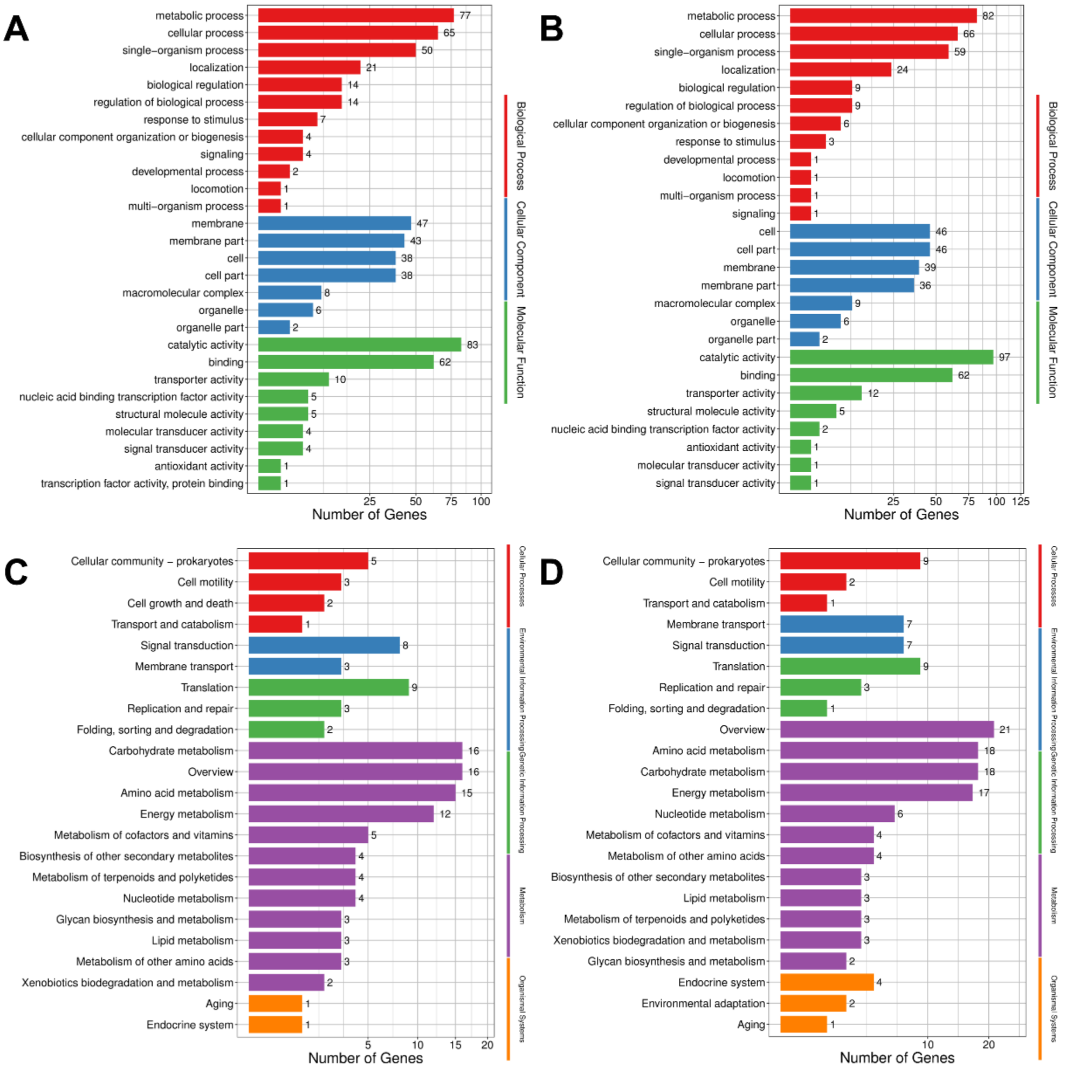

Figure S3 Analysis of CID boundary gene characteristics. (A), (B) GO enrichment results of the CID boundary regions at OD_{600}=0.4 and 1.5, respectively. **(C), (D)** KEGG analysis of the CID boundary regions at OD_{600}=0.4 and 1.5, respectively.

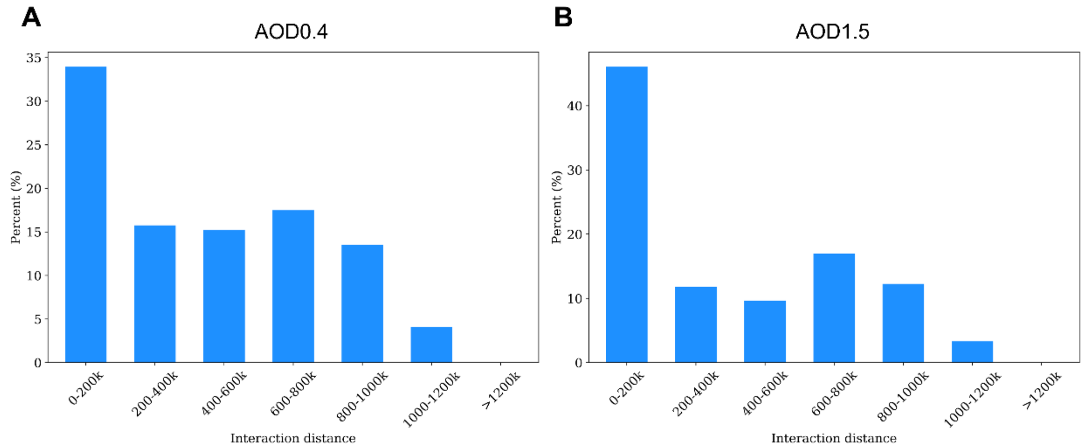

Figure S4 Frequency of significant *cis*-interactions by distance. (A) Frequency of significant *cis*-interactions by distance of *B. melitensis* at OD_{600}=0.4. **(B)** Frequency of significant *cis*-interactions by distance of *B. melitensis* at OD_{600}=1.5. The *cis*-significant interaction loci are classified according to different distance ranges. The interaction distance is processed according to the circular genome. If the distance between the significant interaction loci exceeds half of the length of the chromosome where it is located, the distance between the interaction loci is subtracted from the chromosome length, and the interaction distance is taken as the interaction length. The x-axis is the interval of different interaction distances; the y-axis is the proportion of significant interactions within the distance interval to the total significant interactions.

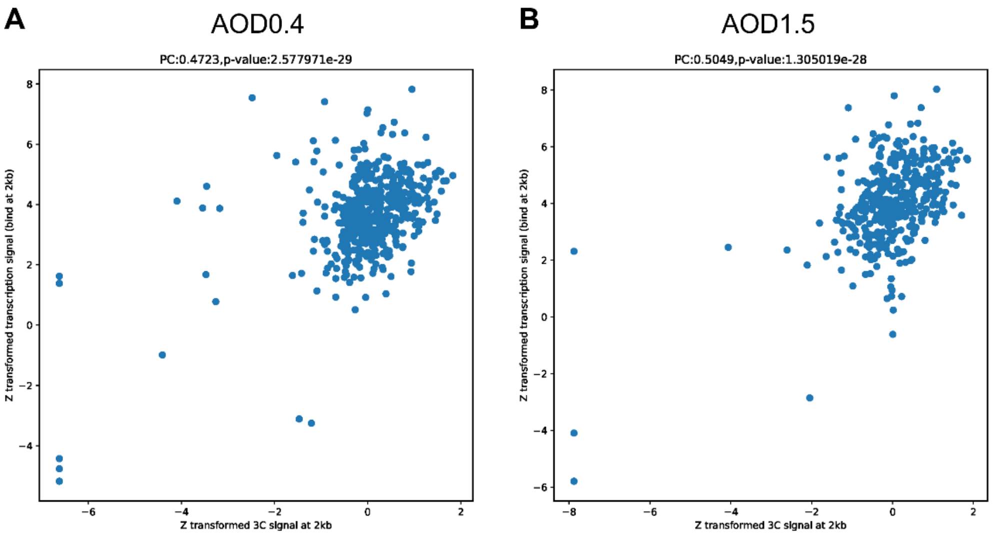

Figure S5 Scatter plots between 3C signal and gene transcription level of AOD0.4 and AOD1.5, respectively. (A) Scatter plot between 3C signal and gene transcription level of of *B. melitensis* at OD_{600}=0.4. **(B)** Scatter plot between 3C signal and gene transcription level of of *B. melitensis* at OD_{600}=1.5. The x-axes represent the Z transformed 3C signal at 2 kb. The y-axes represent the Z transformed transcription signal at 2 kb.

Table S1 The number and length distribution of CIDs

Sample	CID Number	Min	Median	Mean	Max
AOD0.4	79	12,000	38,000	41,708	106,000
AOD1.5	82	16,000	35,000	40,182	94,000

Note:
1) CID Number: The CID number of sample.
3) Min、Median、Mean、Max: The length distribution (kb) of CIDs.

Table S2 Gene density analysis of CID

Sample	CID Number	Min	Median	Mean	Max	Wilcox.test
AOD0.4 border	227	1	3	3	6	4.24e-01
AOD0.4 interior	3166	1	3	3	6	
AOD1.5 border	236	1	3	3	6	3.65e-01
AOD1.5 interior	3162	1	3	3	6	

Note:
1) CID Region: The CID region is divided into border and interior.
2) Gene Number: The number of genes within the CID region.
3) Min、Median、Mean、Max: Gene distribution of CID.

Table S3 GC content distribution of CID regions

CID region	Bin Number	Min	Median	Mean	Max	Wilcox.test
AOD0.4 border	77	49.15	56.8	56.89	61.2	1.06e-01
AOD0.4 interior	1571	43.1	57.6	57.24	64.3	
AOD1.5 border	80	46.4	57.1	56.78	61.2	9.57e-02
AOD1.5 interior	1568	43.1	57.55	57.24	64.3	

Note:
1) CID Region: CID region is divided into border (Border) and internal (Interior).
2) Bin number: the number of bins in the CID region.
3) Min, Median, Mean, Max: GC content distribution of CID.
4) Wilcox.test: wilcoxon test of CID boundary and internal GC content distribution.

Table S4 Motif enrichment information for CID boundaries

sample	motif_ID	percent	border number contained motif	all border number	motif_alt_id
AOD0.4	MX000111	62.34	48	77	RpoN
AOD0.4	MX000022	62.34	48	77	CRE
AOD0.4	MX000208	58.44	45	77	PrrA
AOD1.5	MX000208	66.25	53	80	PrrA
AOD1.5	MX000022	58.75	47	80	CRE
AOD1.5	MX000111	56.25	45	80	RpoN

Note:
1) Samples: sample name.
2) motif_ID: the ID of the motif.
3) percent: The ratio of the boundary containing this motif to the total boundary.
4) border number contained motif: the number of borders containing this motif.
5) all border number: the number of all borders.
6) motif_alt_id: motif alias.

Table S5 Statistical results of significant interaction loci with 1-kb resolution

sample	Significant *cis*-interactions	Significant *trans*-interactions
AOD0.4	49,363	59,953
AOD1.5	25,965	35,938
